# IGFBP7 is upregulated in islets from T2D donors and reduces insulin secretion

**DOI:** 10.1016/j.isci.2024.110767

**Published:** 2024-08-20

**Authors:** Efraim Westholm, Alexandros Karagiannopoulos, Nicole Kattner, Yara Al-Selwi, George Merces, James A.M. Shaw, Anna Wendt, Lena Eliasson

**Affiliations:** 1 Islet Cell Exocytosis, Lund University Diabetes Centre (LUDC), Department of Clinical Sciences-Malmö, Lund University, Malmö, Sweden; 2 Clinical Research Centre (CRC), Skåne University Hospital, Malmö, Sweden; 3 Translational and Clinical Research Institute, Newcastle University, Newcastle upon Tyne, UK; 4 Image Analysis Unit, Newcastle University, Newcastle upon Tyne, UK

**Keywords:** Physiology, Cellular physiology, Cell biology

## Abstract

Intra-islet crosstalk has become a focus area to fully understand the regulation of insulin secretion and impaired β-cell function in type 2 diabetes (T2D). Here, we put forward evidence for insulin-like growth factor binding protein 7 (IGFBP7) as a potential protein involved in autocrine and paracrine β-cell regulation. We showed presence of IGFBP7 in granules of both human α- and β-cells and measured elevated gene expression as well as IGFBP7 protein in T2D. Insulin secretion was reduced in human islets, and the human β-cell line EndoC-βH1, after 72-h incubation with IGFBP7. Mechanistically reduced insulin secretion by IGFBP7 is attributed to reduced p21-activated kinase 1 (PAK1) protein, and decreased oxygen consumption and ATP-production. Knockdown of IGFBP7 in EndoC-βH1 cells verified reduced IGFBP7 levels in the medium, as well as improved insulin secretion. Finally, IGFBP7 knockdown in islets from T2D donors improved insulin secretion, making IGFBP7 a potential drug target in diabetes.

## Highlights

• IGFBP7 gene and protein is elevated in islets from donors with type 2 diabetes
• IGFBP7 is elevated in more in α-cells than β-cells in type 2 diabetes
• IGFBP7 decreases insulin secretion by impairing mitochondrial function
• Knockdown of IGFBP7 improves insulin secretion in islets from dysglycemic donors

## Graphical abstract

**Figure:**
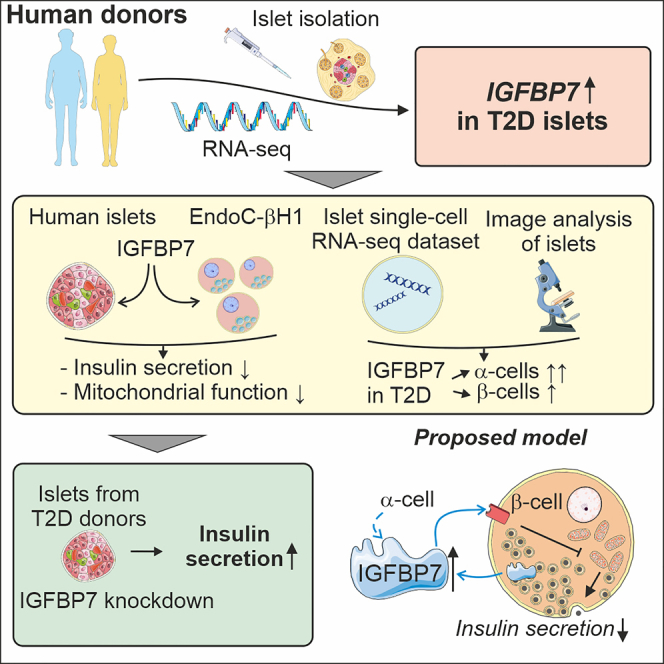

## Introduction

Type 2 diabetes is the most common metabolic disease in the world, affecting the lives of more than 500 million people globally.^1^ Currently, the main view of type 2 diabetes development suggests that insulin resistance in target tissues such as liver and muscle in combination with decreased insulin secretion leads to hyperglycemia.^2^ Deterioration of β-cell insulin secretion has been shown to precede the progression from pre-diabetes to type 2 diabetes.^3^ The regulation of insulin secretion is tightly controlled by several mechanisms.^4^ Knowledge of the stimulus-secretion coupling pathway for insulin secretion from β-cells is key to finding drug-targetable mechanisms to improve insulin secretion. Components of this pathway which may ‘fail’ in diabetes include the glucokinase conversion of the glucose taken up via glucose transporters, the subsequent mitochondrial metabolization of glucose and the fundamental increase in cytosolic ATP:ADP ratio, as well as the Ca^2+^ dependent exocytosis of insulin granules. Moreover, intra-islet crosstalk is essential for paracrine regulation of the β-cells from neighboring islet α- and δ-cells and autocrine regulation from the β-cell itself.^4^^,^^5^

Insulin-like growth factor binding protein 7 (IGFBP7) is a member of the IGFBP-family consisting of seven insulin-like growth factor (IGF)-binding proteins. IGFBP proteins are predominantly secreted by the liver and have been studied as regulators of IGF-1 availability and for their potential involvement in the development of metabolic disorders.^6^^,^^7^ IGFBP7 stands apart from the other IGFBPs by exhibiting higher binding affinity for insulin than for IGF-1 or IGF-2^8^^,^^9^ and IGFBP7 has been shown to enhance the action of insulin at the insulin receptor in liver^10^ as well as interacting with the IGF-1 receptor.^11^ Both IGF-1 and insulin receptor belong to the family of Receptor tyrosine kinase (RTK).^12^ Interestingly, upregulation of the *IGFBP7* gene has also been linked to β-cell maturation.^13^ Insulin resistance is associated with increased circulating levels of IGFBP7 in non-diabetic men^14^ and the *IGFBP7* gene in whole blood samples displays differential DNA-methylation in men recently diagnosed with type 2 diabetes.^15^ IGFBP7 together with tissue metalloproteinase 2 (TIMP2) has been extensively studied as a putative biomarker of kidney failure secondary to both diabetes and ischemia,^16^^,^^17^ and in heart failure.^18^ Its documented interaction with insulin^8^ and the insulin receptor^10^ makes it highly interesting to study in the context of type 2 diabetes and islet function. However, nothing is known about the effects of IGFBP7 on insulin secretion in type 2 diabetes, or if IGFBP7 could be a therapeutic target to improve insulin secretion in clinical type 2 diabetes.

Here, we aimed at investigating the role of IGFBP7 in human pancreatic islets with a special focus on insulin secretion. We also explored how manipulation of IGFBP7 levels impact the function of type 2 diabetes human donor islets.

## Results

### *IGFBP7* is upregulated in pancreatic islets in type 2 diabetes

**Figure 1:**
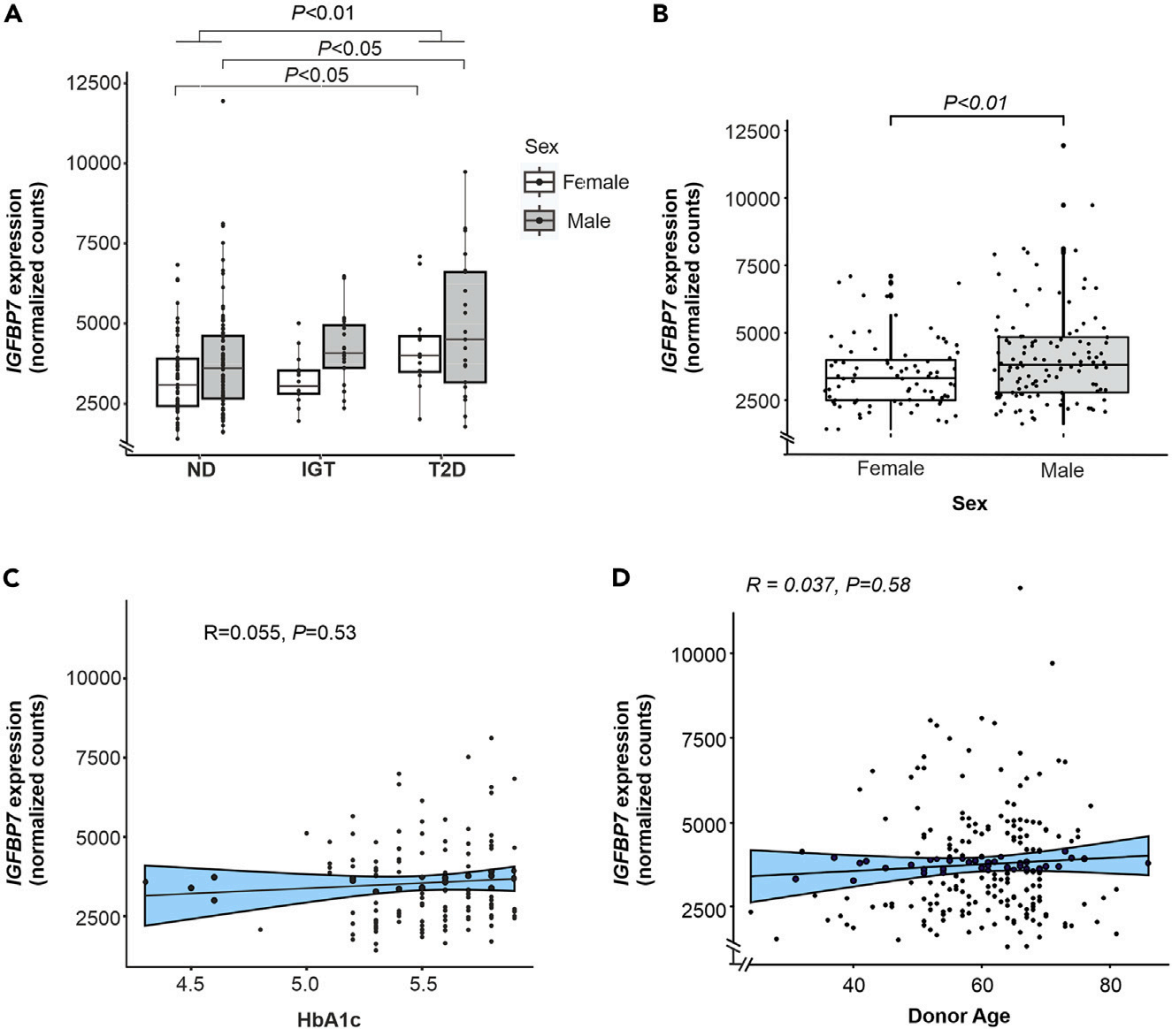
*IGFBP7* is upregulated in pancreatic islets in type 2 diabetes (A) *IGFBP7* gene expression in islets from donors with ND (non-diabetic) and donors with missing HbA1c value, IGT (impaired glucose tolerance) and T2D (type 2 diabetes), as indicated. ND: HbA1c < 42; *N* = 149 (F/M: 54/95), IGT 42 ≤ HbA1c < 48; *N* = 37 (F/M: 14/23), T2D, diagnosed with T2D; *N* = 33 (F/M: 12/21). (B) *IGFBP7* gene expression in islets from female (*N* = 80) and male (*N* = 139) donors. (C) Spearman correlation of *IGFBP7* gene expression to HbA1c in pancreatic islets from non-diabetic human donors. Blue shaded area represents 95% CI of the correlation coefficient. Due to missing HbA1c value in 16 out of 149 available donors, these were excluded from this correlation analysis. (D) Spearman correlation of *IGFBP7* expression to age in 219 human donors. Blue shaded area represents 95% CI of the correlation coefficient. Gene expression values represent normalized DESeq2 counts. Mann-Whitney test in (A and B), boxplots show median and 25th and 75th percentiles and whiskers show 1.5∗IQR unless data points fall outside this range, in which case the whisker is drawn to the last point within range. Data in (A, B, C and D) is from Bacos et al.^19^ See also Figure S1 and Table S1.

We first assessed the expression of *IGFBP7* in human pancreatic islets in a previously published dataset.^19^ *IGFBP7* was among the most highly expressed *IGFBP* genes (Figure S1A). *IGFBP7* gene expression was overall elevated in islets from donors with type 2 diabetes. This was true in both female and male donors (Figure 1A) although male donors had a higher IGFBP7 expression in general (Figure 1B). However, *IGFBP7* expression was unrelated to glycemic levels, as a correlation analysis between *IGFBP7* and HbA1c in non-diabetic donors, with glycemic levels within the normal range, showed no association (Figure 1C). Donors with type 2 diabetes were excluded from this analysis, as some of them were prescribed HbA1c-lowering therapies. Using a general linear model for assessing *IGFBP7* and HbA1c correlation in non-diabetic donors with adjustments for age, sex and BMI, *IGFBP7* was still not associated with HbA1c (*p=*0.424). Spearman correlation for age and *IGFBP7* in all donors showed no significant association (Figure 1D). We also assessed expression levels of *IGFBP1-6* in different glucose tolerance groups (Figure S1B). In addition to *IGFBP7* we also found *IGFBP2* and *6* to be upregulated in type 2 diabetes. IGFBP2 has earlier been shown to be associated with insulin resistance^20^ and IGFBP6 is elevated in serum of patients with type 1 diabetes.^21^

To investigate potential regulation of *IGFBP7*, we used the Pscan transcription factor database^22^ which identified *Runt-related transcription factor 1* (*RUNX1*) as a top predicted transcription factor for *IGFBP7* (Table S1). In macrophages *RUNX1* has been identified in promoting cardiac atherosclerosis in diabetes^23^ as well as promoting renal fibrosis.^24^ Taken together, our data suggest that *IGFBP7* is differentially expressed in islets in type 2 diabetes and that *IGFBP7* may be regulated by *RUNX1.*

### IGFBP7 reduces insulin secretion in human islets and downregulates p21-activated kinase 1 (PAK1)

**Figure 2:**
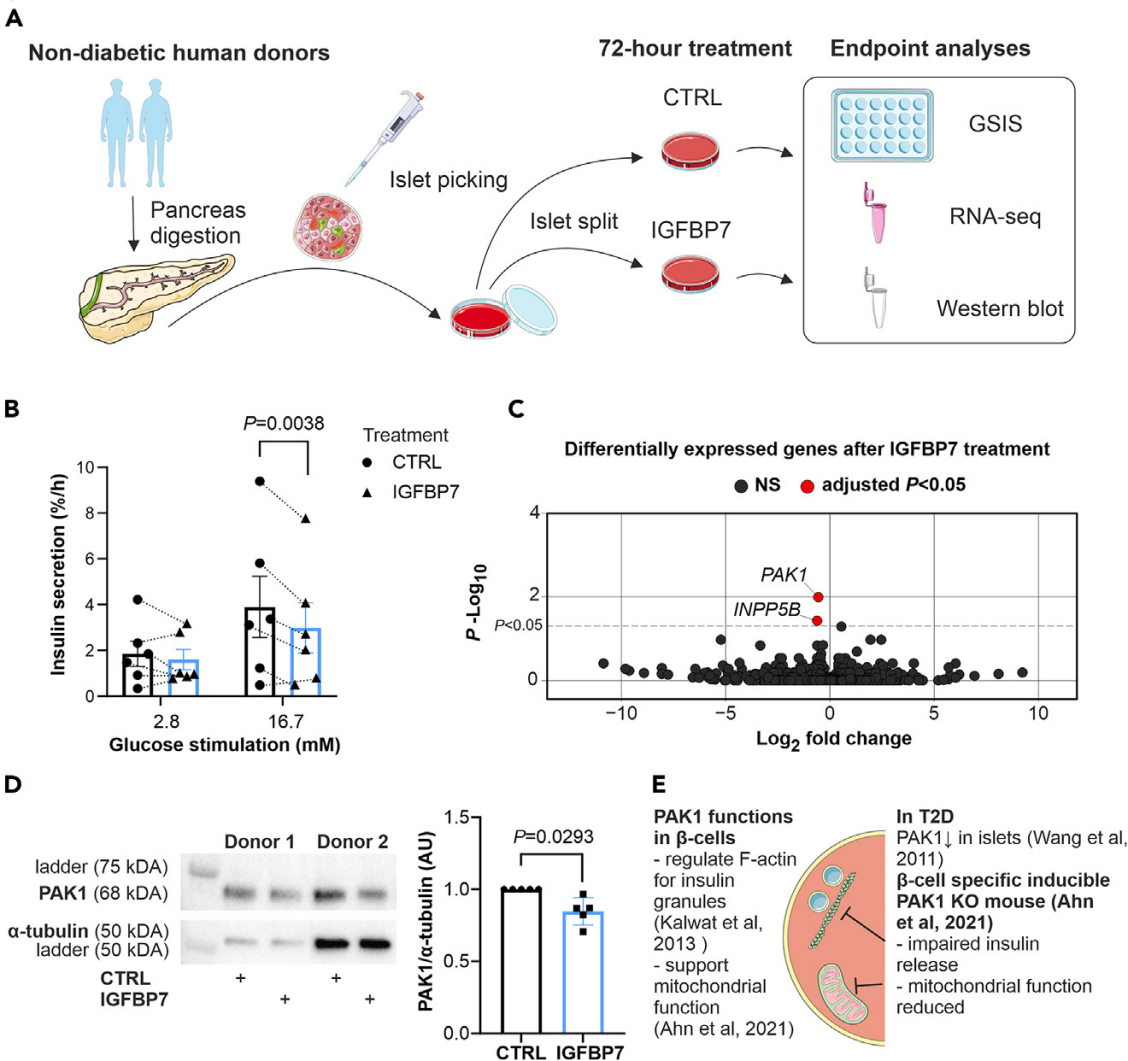
IGFBP7 reduces glucose-stimulated insulin secretion and downregulates PAK1 (A) Schematic workflow of human islet handling and experiments performed. (B) Insulin secretion assay performed in islets from ND human donors after 72-h treatment with (IGFBP7) or without (CTRL) 100 nM IGFBP7 as indicated. Insulin secretion was measured after 1h in 2.8- or 16.7-mM glucose, as indicated (*N* = 6). The dotted lines combine responses from the same individual donors. (C) Volcano plot showing significantly downregulated genes after whole-islet RNA-seq analysis after IGFBP7 treatment. Dashed line indicates the significance threshold of adjusted *p*-value <0.05. Based on data from *N* = 6 CTRL and *N* = 6 IGFBP7 treated islets (as in B). (D) Western blot analyses of PAK1 in CTRL and IGFBP7 treated human islets (same islets as in B) as indicated. Examples from two donors to the left and summary of all experiments to the right (*N* = 5 donors; one of the donors was removed after outlier testing). (E) Summary of known PAK1 functions in β-cells and changes in T2D. In (B), two-way RM ANOVA is used, in (D), paired t-test is used. In (B and D) data are presented as mean ± SEM. Images in (A and E) are from Servier Medical Art, a free unlicensed website for medical images (website URL: https://smart.servier.com/). See also Figure S2 and Tables S2 and S3.

Next, we assessed islet function in human islets incubated for 72 h in 100 nM IGFBP7 (Figure 2A, donor information in Table S2). Glucose-induced insulin secretion was reduced by ∼25% in the IGFBP7-treated islets compared to control, with no difference in basal insulin release (Figure 2B). Insulin content was comparable in the two groups (Figure S2A). To investigate if IGFBP7 alters gene expression, we performed whole-islet RNA-seq. Despite having limited statistical power due to high inter-donor variability, we found two genes that were significantly downregulated following incubation of human islets with IGFBP7: *INPP5B* (inositol polyphosphate 5-phosphatase B) and *PAK1* (p21-activated kinase 1) (Figures 2C; Table S3). INPP5B is implicated in regulating F-actin remodeling in kidney tubular cells^25^ and is associated with regulation of AKT signaling.^26^ The serine/threonine kinase PAK1 has been extensively studied in islet and β-cell physiology for its role in priming of insulin granules via F-actin remodeling,^27^ cell survival,^28^ and supporting mitochondrial function.^29^ PAK1 is downregulated in islets from type 2 diabetes donors,^30^ which prompted us to follow up PAK1 in human islets. Indeed, we could confirm that IGFBP7 treatment reduced PAK1 protein levels by 15% in ND islets (Figure 2D), supporting the proposition that IGFBP7 can reduce insulin secretion through impairment of PAK1 functions (Figure 2E).

### IGFBP7 reduces mitochondrial function in insulin-secreting EndoC-βH1 cells

**Figure 3:**
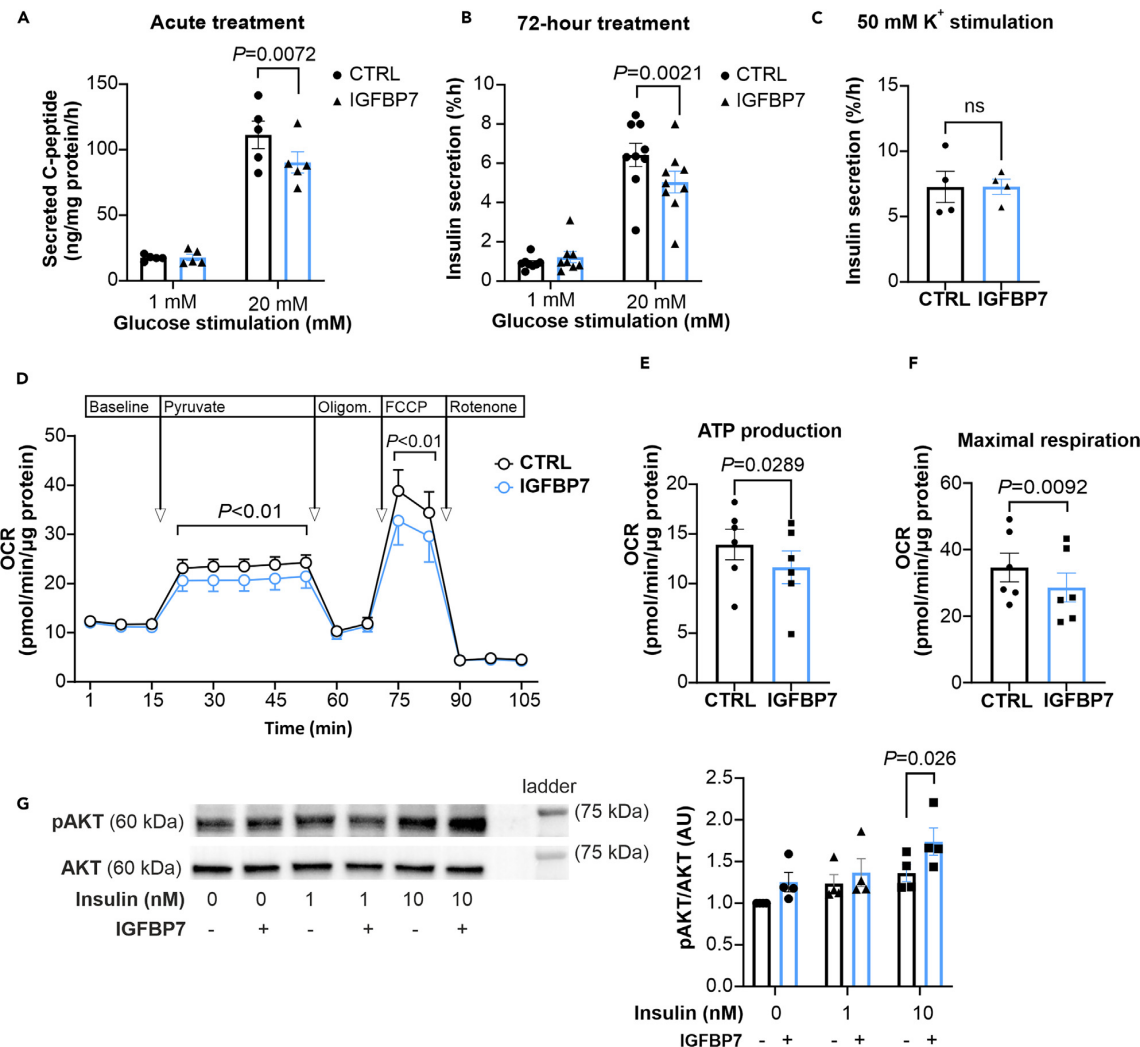
IGFBP7 reduces mitochondrial function in insulin secreting EndoC-βH1 cells (A) Acute 1h treatment with IGFBP7 during insulin secretion assay at 1- and 20-mM glucose in EndoC-βH1 cells (*n* = 3, *N* = 5). (B) Insulin secretion assay performed in EndoC-βH1 cells after 72-h treatment in the absence (CTRL) and presence (IGFBP7) of IGFBP7. Insulin secretion was measured at 1 mM and 20 mM glucose (N = 8–9) as indicated. (C) Insulin secretion assay performed after treatment as in B, but insulin secretion was stimulated with 50 mM KCl (*N* = 4). (D) Oxygen consumption rate (OCR) measurements at baseline and after stepwise injections of pyruvate, oligomycin, carbonyl cyanide *p*-trifluoromethoxy-phenylhydrazone (FCCP) and rotenone, as indicated. Experiments were performed on EndoC-βH1 cells treated in absence (CTRL) or presence of IGFBP7, as indicated. (E and F) ATP production (E) and Maximal respiration (F) calculated from the OCR experiment in D (*N* = 6). (G) Phosphorylation of AKT at Ser473 (pAKT) normalized to AKT after co-treatment with insulin at 0, 1 and 10 nM and IGFBP7 at 100 nM (*N* = 4). In (A, B, D, and G), two-way RM ANOVA is used. In (C, E, and F) paired t-test is used. Data are presented as mean ± SEM. See also Figure S2.

We next used the human clonal β-cell line EndoC-βH1 to further investigate the role of IGFBP7 in β-cells. Initially, we observed that acute treatment (1h) with IGFBP7 resulted in reduced glucose-induced C-peptide secretion (Figure 3A). C-peptide was used as a proxy for secreted insulin in these experiments as IGFBP7 can bind insulin itself.^8^ Long-term effects of IGFBP7 in EndoC-βH1 cells recapitulated those in human islets with a reduction in insulin secretion of ∼20% and no change in insulin content (Figures 3B and S2B), confirming EndoC-βH1 cells as a good model for in depth characterization of IGFBP7 effects on β-cells. Depolarization-induced insulin secretion after long-term treatment with IGFBP7 was not altered (Figure 3C), indicating that IGFBP7 affects processes in the stimulus-secretion coupling pathway upstream of the K_ATP_ channel.^5^ We therefore assessed mitochondrial respiration and confirmed that IGFBP7 treatment reduces the oxygen consumption rate (Figure 3D), consistent with a loss of PAK1 function.^29^ ATP production, measured as oligomycin-linked respiration and maximal respiration was also reduced (Figures 3E and 3F). Moreover, we observed decreased cell viability with increasing concentrations of IGFBP7 (Figure S2C). As cell viability is connected to overall metabolic activity, these results correspond with the observed reduced oxygen consumption rate (Figure 3D).

IGFBP7 interacts with the insulin receptor^8^^,^^10^ and we therefore investigated if IGFBP7 affects insulin receptor signaling in EndoC-βH1 cells. Acute co-treatment with insulin and IGFBP7 resulted in an increased Ser473AKT phosphorylation compared to insulin alone, indicating that IGFBP7 increases the activation of the insulin receptor in β-cells (Figure 3G).

### IGFBP7 is upregulated in α-cells in islets from type 2 diabetic donors

**Figure 4:**
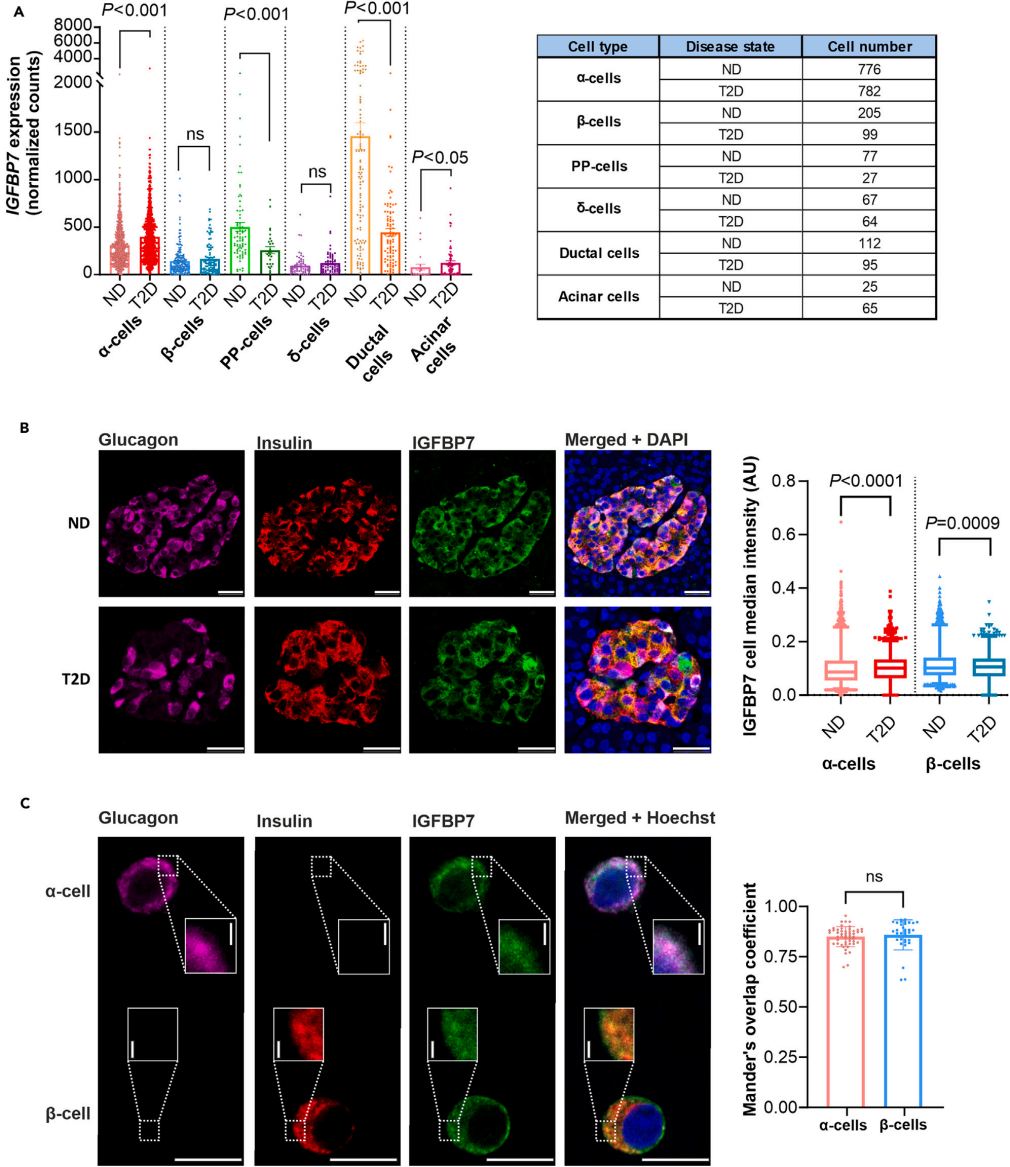
IGFBP7 is upregulated in α-cells in islets from diabetic donors (A) (*Left*) *IGFBP7* gene expression in scRNA-seq of pancreatic endocrine and exocrine cells from 6 ND and 6 T2D donors, as indicated. (*Right*) Table with all cell numbers per cell type and disease state.^31^ (B) (*Left*) Example of immunostaining in one islet from an ND donor and one islet from a T2D donor. Glucagon (pink), insulin (red), IGFBP7 (green) and merge with DAPI nuclear stain (blue). Scale bars 25 μm. (*Right)* Quantification of cell median IGFBP7 intensity in α- and β-cells from six ND and three T2D donors (N: α-cells: ND 5856, T2D 2497; β-cells: ND 3500, T2D 1149). Median values: α-cells: ND 0.0863, T2D 0.102; β-cells: ND 0.102, T2D 0.1059. (C) (*Left*) Example of immunostaining performed in dispersed islet cells from one ND donor. Glucagon (pink), insulin (red), IGFBP7 (green) and merge with Hoechst nuclear stain (blue). Scale bars 10 μm, inset scale bars 1 μm. (*Right*) Quantification using Mander’s overlap coefficient of IGFBP7 and glucagon/insulin in α- and β-cells (N: 52 α-cells, 34 β-cells). Mann-Whitney test in (A and B). In (C), boxplots show median, 25th and 75th percentile and whiskers show 2.5–97.5th percentiles. Student’s *t* test in C. Data in (A and C) are presented as mean ± SEM. See also Figure S2 and Table S2.

To understand how IGFBP7 levels are regulated in the islet, we investigated how *IGFBP7* expression differs across cell types in the pancreatic islets. In Figure 4A, single-cell RNA-seq (scRNA-seq) data on *IGFBP7* from dispersed islet cells from six ND and six type 2 diabetes donors from a published dataset^31^ is presented. In α-cells, *IGFBP7* expression is upregulated by ∼30% in type 2 diabetes donors compared to control whereas the levels in β-cells remain unchanged. Interestingly, *IGFBP7* is highly, albeit variably, expressed in pancreatic ductal cells from ND donors. In type 2 diabetes donors this expression is reduced by 70%. A similar shift with lower *IGFBP7* expression in ductal cells, and then an increase in intraparenchymal cells is seen in a cystic fibrosis-related diabetes (CFRD) animal model where IGFBP7 was suggested to mediate pathological intrapancreatic crosstalk.^32^

In order to investigate IGFBP7 expression in islet cells at the protein level, we immunostained pancreatic sections from ND donors and type 2 diabetes donors for IGBFP7, glucagon, and insulin (for donor information, see Table S2). We then developed an in-house image processing pipeline using guided machine learning to analyze staining intensities of IGFBP7 co-localized with glucagon (α-cells) or insulin (β-cells; Figure 4B). With this approach, we confirmed that the IGFBP7 intensity was increased by ∼20% in α-cells from type 2 diabetes donors compared to control. Also, β-cells exhibited a more marginal but still significant increase in IGFBP7 in type 2 diabetes donors (Figure 4B).

Finally, we characterized IGFBP7 sub-cellular localization in human islets (Figure S2D) and dispersed islet cells (Figure 4C). IGFBP7 co-localizes with both glucagon in α-cells (Mander’s overlap coefficient (MOC): 0.85) and with insulin in β-cells (MOC: 0.86; Figure 4C) indicating that IGFBP7 is enriched in, and possibly released from, large dense-core vesicles together with the primary islet hormones.

### IGFBP7 is released from β-cells and its reduction improves insulin secretion

**Figure 5:**
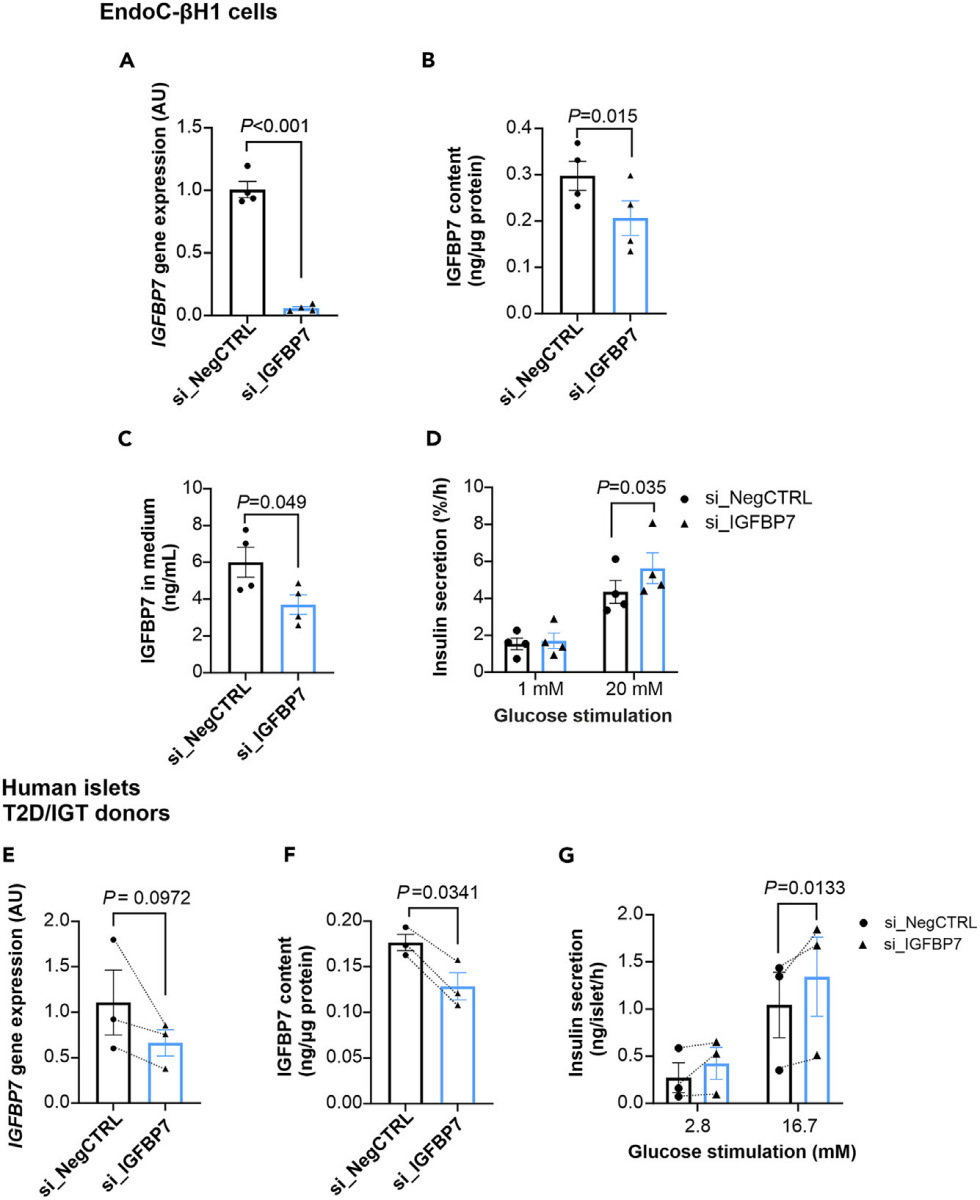
*IGFBP7* knockdown improves insulin secretion (A) *IGFBP7* expression after siRNA-mediated knockdown of *IGFBP7* (si_IGFBP7) in EndoC-βH1 cells compared to control (si_NegCTRL) (*N* = 4). (B) Intracellular IGFBP7 levels after knockdown as in A. (C) IGFBP7 levels in the cell culture medium after knockdown as in A. (D) Insulin secretion at 1 mM and 20 mM glucose after IGFBP7 knockdown (si_IGFBP7) as compared to negative control (si_NegCTRL) (*N* = 4). (E) As in A but experiments were performed in human islets from T2D/IGT donors (*N* = 3). (F) IGFBP7 protein concentrations measured in islets from E. (G) Insulin secretion in human islets from three T2D/IGT donors at 2.8 mM and 16.7 mM glucose after IGFBP7 knockdown (si_IGFBP7) as compared to negative control (si_NegCTRL) (*N* = 3). Paired t-test in (A, B, C, E, and F), two-way RM ANOVA in (D and G). All data are presented as mean ± SEM. See also Figure S2 and Table S2.

To assess if manipulation of endogenous IGFBP7 affects β-cell physiology, we knocked down *IGFBP7* using siRNA in both EndoC-βH1 cells and in islets from three human donors with type 2 diabetes/IGT. In EndoC-βH1 cells we achieved a knockdown of ∼90% (Figure 5A) concomitant with a decrease in IGFBP7 intracellular protein (Figure 5B). IGFBP7 knockdown led to improved glucose-stimulated insulin secretion (Figure 5D) without any changes in insulin content (Figure S2E). Interestingly, reduction of intracellular IGFBP7 was mirrored by a decrease of IGFBP7 in the cell culture medium (Figure 5C) providing support for the hypothesis that IGFBP7 is released from islet cells and can function as an autocrine/paracrine signaling molecule.

Knockdown of IGFBP7 in islets from three donors with impaired glycemic control (Figures 5E and 5F), led to a significant enhancement of insulin secretion in these islets by ∼30% (Figure 5G) with no difference in insulin content (Figure S2F). Taken together, our data indicate that reduction of IGFBP7 levels in the islet can improve β-cell function.

## Discussion

**Figure 6:**
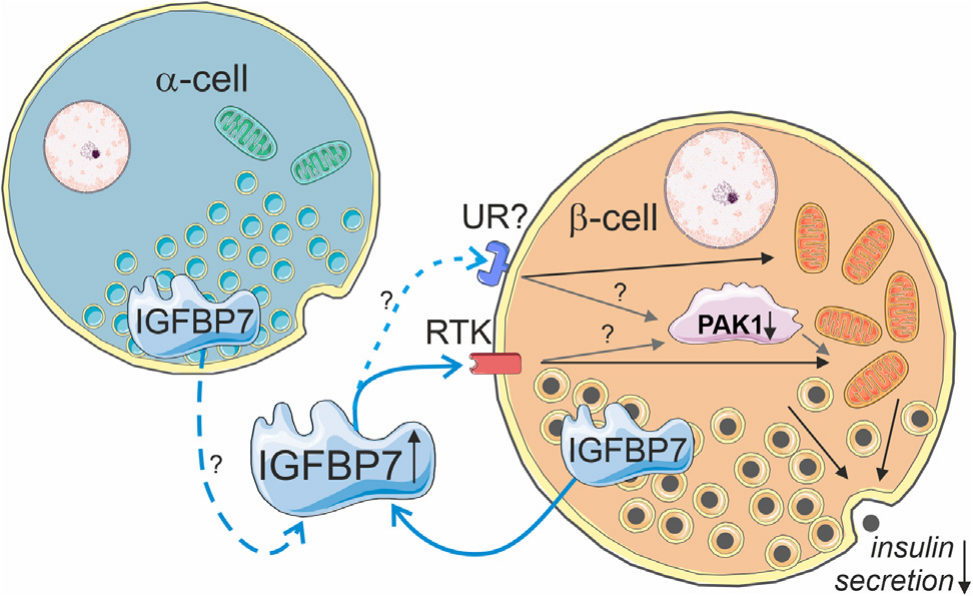
Suggested model for IGFBP7 crosstalk in pancreatic islets Insulin-like growth factor binding protein 7 (IGFBP7) is expressed in both α-cells (*Left*) and β-cells (*Right*). Secreted IGFBP7 interacts with receptor tyrosine kinase (RTK) most likely the insulin receptor. We hypothesize that this leads to a reduction of p21-activated kinase 1 (PAK1) levels. Lower PAK1 levels reduces mitochondrial function, which leads to reduced insulin secretion. An unidentified receptor (UR) has been added to indicate the possibility that IGFBP7 works through another receptor. Image was made using Servier Medical Art, a free unlicensed website for medical images (website URL: https://smart.servier.com/).

Here we have, for the first time, characterized IGFBP7 in human pancreatic islets with focus on expression levels in type 2 diabetes and potential function of this insulin binding protein.^8^ We present evidence that IGFBP7 is a negative regulator of insulin secretion and is upregulated in type 2 diabetes. Our findings support the hypothesis that IGFBP7 is released locally from islet cells and exerts its effects by binding to its receptor on β-cells. Our data further suggest that extra-cellular IGFBP7 reduces the expression of PAK1 resulting in impaired mitochondrial function (Figure 6 for suggested model). Finally, we show that reduction of islet IGFBP7 in type 2 diabetes results in improved insulin secretion.

IGFBP7 is present in human serum/plasma in the low nanomolar range^18^^,^^33^ and is elevated in the serum of newly diagnosed men with type 2 diabetes.^14^ However, it is unclear if the level of IGFBP7 in serum is high enough to affect β-cells directly. Other, more localized, sources are possible and in the CFRD ferret model with impaired insulin secretion, *IGFBP7* is upregulated in parenchymal cells surrounding the pancreatic islets cells.^32^ Indeed, our experiments indicate that IGFBP7 secreted within the islet contributes to IGFBP7 regulation of insulin secretion. The concentrations we used in our experiments were 10–100 times higher than circulating serum concentrations. In a dose-finding experiment with a treatment range of IGFBP7 from 5 to 100 nM in EndoC-βH1 cells, a slight decrease in insulin secretion was observed already at 10 nM with a more pronounced decrease at 100 nM (*data not shown*). Locally secreted IGFBP7 can feasibly reach substantially greater tissue concentrations than the serum concentrations. We suggest that local secretion from the islet cells themselves is the main source of IGFBP7-related regulation (Figure 6). Indeed, IGFBP7 is expressed in both α- and β-cells and we showed that IGFBP7 is secreted from the β-cells.

We found that chronic exposure to IGFBP7 impaired glucose-stimulated insulin secretion but not depolarization induced insulin secretion or insulin production, indicating a defect upstream of the K_ATP_ channel in the stimulus-secretion coupling pathway. IGFBP7 and insulin have been reported to co-stimulate the insulin receptor in liver organoids^10^ and when testing this possibility in our β-cell model we observed a similar mechanism, where insulin and IGFBP7 co-stimulated an AKT-dependent pathway. AKT can be activated by RTKs such as the insulin- and the IGF-1 receptor^12^ but also by certain G-protein coupled receptors (GPCR).^34^ We cannot rule out that IGFBP7 binds more than one receptor in our study, but the fact that we detect increased AKT activation when IGFBP7 is co-administered with insulin indicates that RTKs are involved in the process. We further show that IGFBP7 reduces levels of the serine/threonine kinase PAK1 with a concomitant reduction in mitochondrial function. The molecular mechanism behind the PAK1 reduction is not clear. In *Caenorhabditis elegans* it has been reported that activation of DAF2 (corresponding to the insulin receptor in mammals) leads to cytoplasmic retention of the FOXO equivalent DAF-16 via pAKT activation and reduced transcription of PAK1.^35^ Interestingly, PAK1 has previously been shown to support mitochondrial function through maintaining proper electron transport chain protein expression.^29^ In further support of our data, PAK1 is downregulated in islets from type 2 diabetes donors^30^ and knockdown of PAK1 in EndoC-βH1 cells reduces ATP production and insulin secretion,^29^ similar to our observations with IGFBP7. Mitochondrial ATP production in response to glucose is important in insulin release through closure of the K_ATP_ channel.^36^ Furthermore, the association between dysfunctional mitochondria and diabetes is evident, and several polymorphisms in mitochondrial genes are associated with type 2 diabetes development. Among various mitochondrial perturbations observed in islets from type 2 diabetes donors are diminished ATP:ADP ratio in response to glucose, and reduced β-cell viability.^37^ ATP is also necessary in insulin granule priming^5^ and several other key processes in the β-cell such as cytoskeleton organization for insulin granule transport.^38^

Acute exposure to IGFBP7 also impaired glucose-stimulated insulin secretion. Downregulation of PAK1 is not a likely explanation for the acute effects because of the time aspect, instead we hypothesize that another, probably RTK-dependent, pathway is activated. For instance, the downstream effects of insulin receptor activation (a well-studied RTK) on β-cell insulin secretion is highly context-dependent^39^ and hyperstimulation of the insulin receptor can lead to opening of the K_ATP_ channel,^40^ resulting in reduced insulin secretion.

Importantly, we demonstrate improved/rescued insulin secretion by reducing IGFBP7 in the islets from T2D/IGT donors. We acknowledge that the sample size as well as the achieved reduction is small, and the results needs to be interpreted accordingly. Still, it is intriguing that upregulation of IGFBP7 has been described in several conditions relevant to diabetes development such as: diabetic kidney disease,^16^ heart failure,^18^ and fat storage in the liver.^10^ In these studies, inhibiting or reversing IGFBP7 upregulation led to improved outcomes including reduced blood glucose and less liver fat storage,^10^ and blocked development of cardiac fibrosis.^18^ Given that IGFBP7 is upregulated in human pancreatic islets in type 2 diabetes, and that reversing this improves insulin secretion, IGFBP7 is emerging as a possible multi-tissue drug target in diabetes and other metabolic disorders.

In conclusion, our data suggest IGFBP7 as a protein involved in intra-islet crosstalk. Moreover, we have shown that IGFBP7 (1) is upregulated in islets in type 2 diabetes and (2) negatively regulates β-cell function by impairing mitochondrial function and insulin secretion. Moreover, reducing IGFBP7 level improves β-cell function. Hence, controlling IGFBP7 levels could be an important strategy for improved health in metabolic disease.

### Limitations of the study

All studies have limitations, as does this one. A major drawback is the limited availability of and general inter-donor variability present in human islet donor material. To counter this, we have used previously published data from human donors to further characterize *IGFBP7* in a larger cohort. We also used machine learning to, in an unbiased fashion, gather single-cell protein expression data for islet hormones and IGFBP7. To meet the challenges of a functional follow-up of IGFBP7 functions in β-cells, we used the human β-cell line EndoC-βH1. A caveat of the EndoC-βH1 cell line is that it is an immortal cell line generated from fetal cells. Although the EndoC-βH1 cells are not perfect, the cell line shares many similarities to adult primary human β-cells^41^ making it a suitable model for studying human β-cell functions.

## Resource availability

### Lead contact

Requests or further information on resources and reagents used in this work will be fulfilled by the lead contact. Please refer to L.E. (lena.eliasson@med.lu.se).

### Materials availability

This study did not generate any new unique reagents.

### Data and code availability

• Lead contact: Requests or further information on resources and reagents used in this work will be fulfilled by the lead contact. Please refer to Lena Eliasson (lena.eliasson@med.lu.se).
• Data availability: previously published islet RNA-seq data and donor characteristics used in Figures 1A–1D, S1A, and S1B can be accessed upon reasonable request to the lead contact of that work.^19^ The human islet RNA-seq dataset with or without treatment with IGFBP7 were deposited in the Lund University Diabetes Center (LUDC) repository (https://www.ludc.lu.se/resources/repository) under the following accession number LUDC IGFBP7 cohort (accession number LUDC2024.08.114), and is available from the lead contact (Lena Eliasson) upon request but access to data must be granted by the LUDC Human Tissue Laboratory steering committee. Individual-level data are not publicly available due to ethical and legal restrictions related to the Swedish Biobanks in Medical Care Act (2023:38) and Personal Data Act (1998:204). Summary statistics describing these data are presented in Table S3. Previously published scRNA-seq data used in Figure 4A can be found at (GEO: GSE153855) and is described in Ngara and Wierup.^31^
• Code availability: Code developed for analyzing IGFBP7 stain intensities in α- and β-cells in images of pancreatic tissue slices is available on Mendeley Data (https://doi.org/10.17632/hwg4hfcykg.1).

## Acknowledgments

We thank Anna-Maria Veljanovska-Ramsay, Eugenia Cordero Concha, Sara Karlsson, Mary Lagoguez, and Caitlin Brack for technical assistance. We thank Olle Korsgren and the Nordic Network for Clinical Islet Transplantation, the tissue isolation teams and Human Tissue Laboratory within EXODIAB/LUDC. We thank Elaine Cowan and Mototsugu Nagao for methodological assistance, Malin Svensson and Fong To at the LUDC Sequencing Facility, Anna-Maria Dutius Andersson at LUDC Confocal Imaging Unit, and the Newcastle University Bioimaging Facility. This work was funded by the UK Cystic Fibrosis Trust (CFRD SRC-019), The Swedish Foundation for Strategic Research (IRC15-0067), the 10.13039/501100004359Swedish Research Council (project L.E. (2019-01406) and Strategic Research Area Exodiab (2009-1039)), The Swedish Diabetes Foundation (L.E.; DIA2022-723), 10.13039/501100004973Barndiabetesfonden, 10.13039/501100006189Albert Påhlsson Foundation, 10.13039/501100009235Riksförbundet Cystisk Fibros, 10.13039/501100005753Royal Physiographic Society in Lund, 10.13039/501100009804Svenska Diabetesstiftelsen. E.W. and N.K. positions are supported by the UK Cystic Fibrosis Trust (CFRD SRC-019). Y.S. PhD position is funded by Newcastle University in partnership with the UK Cystic Fibrosis Trust.

## Author contributions

L.E. supervised the project. E.W., A.W., and L.E. designed the study. E.W., N.K., and Y.S. performed the experiments. A.K. and G.M. performed the gene expression and image analyses, respectively. E.W., A.W., and L.E. wrote the original draft. J.S. gave valuable input and supervised the work in Newcastle. All authors critically analyzed data, reviewed, and edited the manuscript and approved of the final version of the manuscript. L.E. is the guarantor of this work.

## Declaration of interests

The authors declare no relationships or activities related to personal gain, financial or otherwise, that are in a conflict of interest with this work.

## STAR★Methods

### Key resources table

**Table:** 

REAGENT or RESOURCE	SOURCE	IDENTIFIER
**Antibodies**		

Mouse monoclonal anti-glucagon, 1:200	Abcam	Cat# ab10988, RRID: AB_297642
Mouse monoclonal anti-glucagon, clone K79bB10 source # 0000078097, 1:100	Sigma-Aldrich	Cat# G2654, RRID: AB_259852
Guinea-pig polyclonal anti-insulin, 1:200	American Research Products	Cat# 03–16049, RRID: AB_1542133
Flex polyclonal guinea Pig, anti-insulin, 1:5	Dako	Cat# IR002, RRID: AB_2800361
Rabbit polyclonal anti-IGFBP7, 1:200	Origene	Cat# AP01109PU-S, RRID: AB_1617562
Donkey anti-mouse Cy3, 1:400	Jackson ImmunoResearch	Cat# 715-165-151, RRID: AB_2315777
Donkey anti-mouse AF647, 1:500	Invitrogen	Cat# A-31571, RRID: AB_162542
Donkey anti-guinea-pig AF647, 1:400	Jackson ImmunoResearch	Cat# 706-605-148, RRID: AB_2340476
Goat anti-guinea-pig AF568, 1:500	Invitrogen	Cat# A-11075, RRID: AB_2534119
Donkey anti-rabbit AF488, 1:400	Jackson ImmunoResearch	Cat# 711-545-152, RRID: AB_2313584
Donkey anti-rabbit AF488, 1:500	Invitrogen	Cat# A-21206, RRID: AB_2535792
Rabbit polyclonal anti-PAK1, 1:2000	CellSignaling	Cat# 2602, RRID: AB_330222
HRP conjugated goat anti-rabbit, 1:2000	BioRad	Cat# 170–6515, RRID: AB_11125142
Mouse monoclonal anti-α-tubulin, 1:1000	Sigma-Aldrich	Cat# T9026, RRID: AB_477593
HRP conjugated goat anti-mouse, 1:2000	Dako	Cat# P0447, RRID: AB_2617137
Rabbit monoclonal anti-pSer473AKT (pAKT), 1:2000	CellSignaling	Cat# 4060, RRID: AB_2315049
Rabbit monoclonal anti-AKT, 1:1000	CellSignaling	Cat# 4691, RRID: AB_915783

**Biological samples**		

Human pancreatic islets from healthy and T2D diabetic donors	Nordic Network for Clinical Islet Transplantation and Human Tissue Lab at LUDC/EXODIAB	N/A
Human pancreatic tissue slices and pancreatic islets	Quality in Organ Donation, Expand Whole Pancreas Biobank at Newcastle University	N/A

**Chemicals, peptides, and recombinant proteins**		

Recombinant human IGFBP7	Sigma-Aldrich	Cat# GF3066
Human insulin	Novo Nordisk	Actrapid, Nordic medical product ID: 01 35 09
EDTA-free protease inhibitor	Roche	Cat# C762Q77
4-15% Mini Protean TGX Stain-free gel	BioRad	Cat# 4568084
LVPDF membrane	BioRad	Cat# 1704272
Clarity Western ECL substrate	BioRad	Cat# 1706061
4x laemmli sample loading buffer	BioRad	Cat# 1610747
Qiazol lysis reagent	Qiagen	Cat# 79306
OptiMEM reduced serum media	Gibco Life Technologies	Cat# 31985047
Lipofectamine RNAiMAX Transfection Reagent	Thermo Fisher	Cat# 13778075
Sodium pyruvate	Sigma-Aldrich	Cat# P2256
Oligomycin A	Sigma-Aldrich	Cat# 75351
FCCP (carbonyl cyanide 4-(trifluoromethoxy)phenylhydrazone)	Sigma-Aldrich	Cat# C2920
Rotenone	Sigma-Aldrich	Cat# R8875

**Critical commercial assays**		

Human insulin ELISA	Mercodia	Cat# 10-1113-10
Human C-peptide ELISA	Mercodia	Cat# 10-1136-01
Pierce™ BCA Protein Assay Kit	Pierce	Cat# 23225
miRNeasy miRNA/RNA Isolation kit	Qiagen	Cat# 217084
Human IGFBP7 ELISA kit	BoosterBio	Cat# EK0991
Illumina Stranded mRNA Prep ligation kit	Illumina	Cat# 20040532
NSQ 500/550 Hi Output KT v2.5 kit	Illumina	Cat# 20024907
TaqMan Reverse Transcription kit	Thermo Fisher	Cat# 4366597
TaqMan Gene Expression Master Mix	Thermo Fisher	Cat# 4369016
MTS assay, CellTiter 96® AQueous One Solution Cell Proliferation Assay	Promega	Cat# G3582

**Deposited data**		

Whole islet RNA-seq with donor information	Bacos et al.^19^	Available upon request. LUDC2022.07.111 and LUDC2022.07.113
Single-cell RNA-seq with donor information	Ngara M & Wierup N^31^	GEO: GSE153855
Whole islet RNA-seq after IGFBP7 treatment	This paper	Available upon request to the lead contact. LUDC2024.08.114

**Experimental models: Cell lines**		

EndoC-βH1 cells, human fetal clonal β-cell line	Ravassard et al.^42^	RRID: CVCL_L909

**Oligonucleotides**		

Silencer select siRNA targeting IGFBP7:	Invitrogen	Cat# 4392420
Silencer select Negative Control No. 2	Invitrogen	Cat# 4390846
TaqMan gene expression assay IGFBP7	Thermo Fisher	Cat# Hs00266026_m1
TaqMan gene expression assay HPRT1	Thermo Fisher	Cat# Hs04194521_s1
TaqMan gene expression assay PPIA	Thermo Fisher	Cat# Hs02800693_m1

**Software and algorithms**		

GraphPad Prism v. 9	GraphPad	RRID:SCR_002798
CorelDRAW Graphics suite 2021 64-bit	Corel Corporation	RRID:SCR_014235
ImageLab v. 6.1	BioRad	RRID:SCR_014210
Seahorse analytics software	Agilent	RRID:SCR_024491
Zen 3.1 Blue edition confocal image analysis	Carl Zeiss Microscopy	Version: 3.1.0.0000
Leica Application Suite X (LAS X) software	Leica	N/A
Code for analyzing pancreas section images for IGFBP7 stain intensities in different pancreatic islet cell types	This paper.	Mendeley Data: https://doi.org/10.17632/hwg4hfcykg.1

**Other**		

ChemiDoc MP Imager	BioRad	Universal Hood III, 73BR02983
Seahorse XFe24 Analyzer system	Agilent	RRID:SCR_014526
Seahorse XF24 cell culture microplates	Agilent	Cat# 100777004
Carl Zeiss LSM 800 confocal microscope	Carl Zeiss Microscopy	N/A
Leica SP8 STED confocal microscope	Leica	N/A
CLARIOStar microplate reader	BMG Labtech	N/A
Quantstudio Flex7 Real Time quantitative PCR system	Applied Biosystems	Cat# 4485701

### Experimental model and study participant details

#### Ethical approvals

Pancreatic islets used for *in vitro* experiments were obtained through the Exodiab/LUDC collaboration with the Nordic Network for Islet Transplantation. Islets were handled as described in ethical permits given by Etikprövningsmyndigheten, Dnr: 2019–00357. Pancreatic tissue sections from the body area of the pancreas were kindly provided by the Quality in Organ Donation (QUOD) Expand Whole Pancreas Biobank at Newcastle University to perform immunofluorescence staining. Organs were retrieved after written donor family consent in compliance with the UK Human Tissue Act of 2004 and under specific ethical approvals by the UK Human Research Authority (05/MRE09/48).

#### Pancreatic islets for *in vitro* experiments

Donor characteristics and information on use in this study of islets for *in vitro* functional experiments, pancreatic tissue section samples and islets for immunostaining can be found in Table S2. Islets were delivered in CMRL-1066 medium supplemented with 10% human serum. Thereafter, islets were handpicked in KREB’s buffer, split for culture and treatments in RPMI-1640 medium supplemented with: 5 mM glucose, 200 mM L-glutamine, 10% FBS (Sigma-Aldrich, 7524), penicillin and streptomycin 100 U/mL and 100 μg/mL respectively (PAA Laboratories, Pasching, Austria).

#### EndoC-βH1 cell line

EndoC-βH1 cells (EndoC-βH1 cells, Paris, France)^42^ were cultured in culture plates coated with matrigel and fibronectin (100 μg/mL and 2 μg/mL, products from Sigma-Aldrich, Steinheim, Germany). DMEM medium (Thermo Fisher Scientific, Waltham, MA, USA) supplemented with 5.6 mM glucose, 2% BSA (Roche Diagnostics, Mannheim, Germany), 10 mM nicotinamide (Merck Millipore, Darmstadt, Germany), 50 μM β-mercaptoethanol, (5.5 μg/mL transferrin, 6.7 ng/mL sodium selenite (Sigma–Aldrich) and antibiotic mix penicillin and streptomycin 100 U/mL and 100 μg/mL respectively (PAA Laboratories). Cells were kept in a humidified condition at 37°C and CO_2_ levels 5%. Cells were seeded in 48-well plates at a concentration of 900 000 cells/mL, reaching 100% confluence 4 days post seeding. The cell line was regularly tested for mycoplasma contamination.

### Method details

#### Analysis of RNA-seq and scRNA-seq data

For the data generated in this study, total whole-islet RNA was extracted using Qiagen miRNA extraction mini kit (Qiagen, 217084). Libraries were prepared using the Illumina Stranded mRNA Prep, Ligation kit (Illumina, 20040532) and sequenced with the NSQ 500/550 Hi Output KT v2.5 kit (Illumina, 20024907). The quality of the sequence reads was evaluated with FastQC (v0.11.8) and subsequently the reads were mapped to the human genome (Gencode v43) using Salmon (v1.5.2).^43^ Differential expression analysis was performed with DESeq2 (v.1.34)^44^ and reported *p*-values were corrected for multiple comparisons with the Benjamini-Hochberg procedure.

#### IGFBP7 treatment

Human islets were split into 35 mm petri dishes, 500 islets per dish with 1.9 mL RPMI medium. For IGFBP7 treatment, 100 μL IGFBP7 (Sigma-Aldrich, GF3066) stock solution at 2 μM dissolved in 0.1% BSA was added to the dish for a final treatment concentration of 100 nM. As control (CTRL), 0.1% BSA solution was used. EndoC-βH1 cells were seeded in a volume of 190 μL and 24 h post-seeding 10 μL IGFBP7 stock solution was added for a final concentration of 100 nM. Both human islets and EndoC-βH1 cells were incubated for 72 h post-treatment before insulin secretion assays, RNA, and protein sample collection. In experiments with acute treatment, IGFBP7 was added to the secretion assay buffer to a 100 nM concentration.

#### SiRNA knockdown of *IGFBP7*

Transfection complexes for siRNA *IGFBP7* (Invitrogen, 4392420) and siRNA Negative Control (Invitrogen, 4390846) were prepared with RNAiMax transfection reagent (Thermo Fisher, 13778075) in OptiMEM (Gibco Life Technologies, 31985047). Both human islets and EndoC-βH1 cells were transfected twice with a final siRNA concentration of 10 nM. Islets were transfected the day of receival, 24 h post first transfection and assayed for insulin secretion and RNA and protein sample collection 48 h post second transfection for a total treatment time of 72 h. EndoC-βH1 cells were first transfected 24 h post-seeding, then followed the same procedure as for islets.

#### Insulin secretion assay

Human islets were pre-incubated for 30 min in 0.5 mL KREBS buffer supplemented with 2.8 mM glucose. Insulin secretion was then performed in quadruplicate for each condition, with 10 islets handpicked to wells in a 24-well plate. Specifically, islets were serially incubated for 1 h in KREBS buffer with 2.8 mM glucose, and then 1 h in KREBS with 16.7 mM glucose.

Insulin secretion in EndoC-βH1 cells was performed in triplicate for each condition and for each glucose level (1 mM, 20 mM). Confluent EndoC-βH1 plates were pre-incubated with 0.5 mL secretion assay buffer (SAB), pH 7.2 (1.16 mM MgSO_4_, 4.7 mM KCl, 1.2 mM KH_2_PO_4_, 114 mM NaCl, 2.5 mM CaCl_2_, 25.5 mM NaHCO_3_, 20 mM HEPES, and 0.2% bovine serum albumin) containing 1 mM glucose for 2 h. The cells were then stimulated with 0.25 mL SAB containing 1 or 20 mM glucose for 1 h. For both islets and EndoC-βH1 cells: sample for insulin content and total protein from each well was extracted using 100 μL RIPA buffer: 0.1% SDS, 150 nM NaCl, 1% Triton X-100, 50 mM Tris-Cl, pH 8, and EDTA-free protease inhibitor (Roche, C762Q77). For analysis of secreted insulin, insulin content of each well and protein, see section human insulin and C-peptide ELISA and total protein analyses.

#### Seahorse assay

EndoC-βH1 cells were seeded as described in section EndoC-βH1 cell line in Seahorse 24-well plates (Agilent, 100777004) designed for use in the Seahorse XFe24 analyser system with modification for lower well volumes. Treatment with IGFBP7 was performed as described in section IGFBP7 treatment and oxygen consumption rate (OCR) measurements were done 72 h post-treatment. Cells were washed and preincubated 2 h with a modified secretion assay buffer with a glucose concentration at 1 mM (see section insulin secretion assay) without albumin and NaHCO_3_. Mitochondrial OCR was measured at baseline, and then after injections in the following order: 10 mM pyruvate, 5 μg/mL oligomycin, 4 μM carbonyl cyanide *p*-trifluoromethoxy-phenylhydrazone (FCCP), and 1 μM rotenone. Mitochondrial OCR was analyzed using the specified online tool for the analyzer system (URL: seahorseanalytics.agilent.com) and normalized to total protein in each well, see section human insulin and C-peptide ELISA and total protein analyses.

#### RNA extraction and RT-qPCR

Total tissue input for human islets in RNA samples was 100 handpicked islets and for EndoC-βH1 cells two pooled 48-well wells. RNA was collected in Qiazol lysis reagent (Qiagen, 79306) and extracted using the Qiagen miRNeasy mini kit (Qiagen, 217084). cDNA was prepared from 80 ng RNA using TaqMan Reverse Transcription kit (Thermo Fisher, 4366597) and RT-qPCR was performed on a Quantstudio Flex7 (Applied Biosystems). Primer for *IGFBP7*: Hs00266026_m1 (Thermo Fisher) and normalized to the Geomean expression of *HPRT1* and *PPIA* (Thermo Fisher, Hs04194521_s1; Hs02800693_m1) using the 2^-ΔΔCt^ method.

#### Western blot

Protein samples for Western blot analysis of PAK1 levels in human islets were collected in RIPA buffer. Briefly, 100 islets were washed in PBS and centrifuged. PBS was removed and 200 μL RIPA was added. Samples were sonicated and boiled for 10 min at 70°C. Of each sample, 7.5 μL was mixed 1:1 with 4× laemmli sample loading buffer (BioRad, 1610747) and run on a 15-well precast gel (BioRad, 4568084) for 30 min at 200 V, 3 A and 300 W. Stain-free gel images were taken, and protein bands were transferred to an LVPDF membrane (BioRad, 1704272). Stain-free blot images were taken, and the membrane was blocked for 1 h in 3% BSA in TBS-T. Primary antibody rabbit anti-PAK1 (1:2000, CellSignaling, 2602) was dissolved in 1% BSA in TBS-T and incubated at 4°C overnight. Membrane was washed in TBS-T, and secondary antibody against rabbit (1:2000, BioRad, 170–6515) was incubated for 1 h at room temperature. Chemiluminescent images were obtained with Clarity Western blot ECL substrate solution (BioRad, 1706061). Normalization control used for PAK1 was α-tubulin. Membrane was stripped and re-probed with mouse anti-α-tubulin antibody (1:1000, Sigma-Aldrich, T9026), and secondary antibody anti-mouse (1:2000, Dako, P0447).

For experiments on insulin receptor pathway activation for assessment of phosphorylated AKT (pSer473AKT, pAKT), we co-treated EndoC-βH1 with insulin (Novo Nordisk, Actrapid) at 0, 1 and 10 nM together with 100 nM IGFBP7 (Sigma-Aldrich, GF3066) for 15 min. Protein samples were collected by snap-freezing cell plates in liquid nitrogen and thereafter lysing the cells directly in loading buffer. Samples were run on 10-well precast gels (BioRad, 4568084) with the same settings and gel handling as described above. Membranes were then on subsequent days probed for pAKT (pSer473AKT) (1:2000, CellSignaling, 4060) and then AKT (1:1000, CellSignaling, 4691) with secondary antibody (1:2000, BioRad, 170–6515). All images were acquired on a BioRad ChemiDoc MP Imager (Universal Hood III, 73BR02983) and quantified using Image Lab v. 6.1 software (BioRad, RRID:SCR_014210).

#### Immunostaining of dispersed islet cells, pancreatic tissue slices and isolated islets

##### Dispersed islet cells

Approximately 300 islets from one human donor were centrifuged at 150 g for 2 min, washed in PBS, and dissociated with trypsin (Cytiva, SH30236.02) for 8 min in a water bath at 37°C. Dispersed cells were washed with PBS and centrifuged at 300 g for 10 min. Cells were resuspended in RPMI medium to a concentration of ∼1 000 000 cells/mL. Finally, ∼10 000 cells were seeded in a 10 μL droplet in a Nunc LabTek II 8-well chamber (Thermo Fisher, 154534), and rested for 2 h to allow cells to adhere to the well bottom before addition of 300 μL RPMI medium. One day after dispersion, cells were fixated and stained for glucagon, insulin, and IGFBP7. Briefly, seeded dispersed cells were washed with PBS before 5 min fixation with 3% PFA-K-PIPES and then 10 min 3% PFA-Na2BO4. Cells were permeabilized with 0.1% Triton X-100 for 30 min at room temperature and then blocked with 5% normal donkey serum (NDS) (Jackson ImmunoResearch, West Grove, PA, USA) in PBS. Primary antibodies against glucagon (1:200, Abcam, ab10988, RRID: AB_297642), insulin (1:200, Progen, 16049, RRID: AB_1542133), and IGFBP7 (1:200, Origene, AP01109PU-S, RRID: AB_1617562), were diluted in 5% NDS and incubated for 2 h. Fluorescently labeled secondary antibodies (1:400 Cy3, 715-165-151, RRID: AB_2315777; 1:400 AF647, 706-605-148, RRID: AB_2340476; 1:400 AF488, 711-545-152, RRID: AB_2313584, all Jackson ImmunoResearch) were used to detect the primary antibodies. Cells were visualised with immersion oil in 63× magnification using the LSM800 confocal microscopy system (Carl Zeiss, Germany) and Mander’s overlap coefficient was obtained using the colocalization tool in the software Zen Blue 3.1 analysis package (Carl Zeiss).

##### Pancreatic tissue slices and isolated islets

Antigen retrieval was performed with sodium citrate buffer at pH 6 for 20 min at 100°C with cooling to 40°C. Tissue sections or isolated islets were then blocked for 1 h with 20% fetal bovine serum (FBS) in phosphate buffered saline (PBS) at room temperature followed by incubation with primary antibodies overnight at 4°C. Primary antibodies for glucagon (1:100, Sigma-Aldrich, G2654-2ML, RRID: AB_259852), insulin (1:5, Dako, IR002, RRID: AB_2800361), and IGFBP7 (1:200, Origene, AP01109PU-S, RRID: AB_1617562) were diluted in 0.05% FBS in PBS. No primary antibody control sections were used for every donor. The following day, slides were washed three times in PBS for 5 min on a rotating platform, followed by incubation with the secondary antibodies for 1 h at room temperature in the dark. Secondary antibodies Alexa Fluor 647 (1:500, Invitrogen, A31571, RRID: AB_162542), Alexa Fluor 568 (1:500, Invitrogen, A11075, RRID: AB_2534119), and Alexa Fluor 488 (1:500, Invitrogen, A21206, RRID: AB_2534119) were diluted in 0.05% FBS in PBS. Subsequently, the slides were washed three times in PBS for 5 min on a rotating platform and incubated with 4′,6-diamidino-2-phenylindole (DAPI) solution (Thermo Fisher, Paisley, UK) at a concentration of 0.1 mg/mL in PBS for 20 min at room temperature in the dark. The slides were then mounted with VECTASHIELD Antifade Mounting Medium with DAPI (2bscientific, Kirtlington, UK) and sealed with nail polish. All slides were stored at 4°C protected from light prior to imaging on the Leica SP8 STED confocal microscope and analyzed with the Leica Application Suite X (LAS X) software (Leica).

#### Analysis of immunostaining of pancreatic tissue slices

Below you will find an outline of development of an analysis software using guided machine learning to identify islets, individual islet cells, and classification into α-cells or β-cells respectively using glucagon and insulin staining intensities. IGFBP7 staining intensity was analyzed in classified islet cells.

##### Processing of images for analysis

Image stacks were processed using a combination of FIJI,^45^ Ilastik,^46^ and CellProfiler.^47^ An ImageJ macro was developed to perform all processing of the images prior to analysis in CellProfiler.

##### Segmentation of Islet regions

A selection of 11 images were selected at random and partially annotated within Ilastik for regions of islet and non-islet. Once training had reached a visually acceptable accuracy on unlabelled regions of the training images, the model was applied to 5 testing images. Output probability maps from the testing images were then run through a macro to generate thresholded binary images using different pre-processing parameters, to test 1936 different options. These output binary images were compared to manually annotated versions of the testing images to identify the quantitatively more similar options. A manual sense-check is then done of highly similar processes to determine the optimum method. Optimum thresholding parameters were found to be pre-processing by subtracting a pixel value of 40 from the 8-bit probability maps, applying a Gaussian blur with a sigma value of 20 pixels, and thresholding using the Huang algorithm within FIJI. The binary islet region is then inverted and applied as a mask to the nuclear channel to leave only nuclear signal within the islet regions.

##### Nuclear segmentation

A random selection of 8 nuclear channel images from the full dataset (single z points) were manually annotated within FIJI. The images were run through a macro that tests 1701 parameter combinations within the nuclear segmentation tool StarDist.^48^ Output nuclear segmentation options were quantitatively compared to the manual annotation by assessing the number of nuclei detected along with the nearest neighbor distance between the automatically identified nuclei and the manually identified nuclei, followed by a manual sense-check to determine the most similar. The optimal settings for this were found to be conversion to 8-bit format, application of a Gaussian blur with a sigma value of 9 pixels, followed by application of StarDist using a probability threshold of 0.7 and nms threshold of 0.8. The resulting nuclei objects were painted onto a new blank image to allow for single nuclei to be visualised without overlap.

##### Cell boundary determination

Single-cell segmentation was performed in CellProfiler.^47^ Binary nuclear images are used to generate cell seeds, with each nucleus acting as an individual seed. The three non-nuclear image channels are then rescaled to fill the maximum intensity range and combined in a maximum intensity projection. The nuclear seeds are expanded using the maximum intensity projection image to assist in cell boundary determination. Cell expansion is carried out within CellProfiler using the propagation method, using the global minimum cross-entropy thresholding method, with a smoothing factor of 1 and a threshold correction factor of 1. A regularisation factor of 0.25 was used. These parameters were selected by performing segmentation under a variety of conditions and visually assessing outputs for optimum conformity to perceived cell boundaries. Individual cell objects were then assessed for morphological and fluorescence intensity measurements with results exported in a.csv format.

##### Cell classification

Single cell median intensity was recorded for insulin and glucagon fluorescent labeling. The insulin label fluorescent intensity was compared to the glucagon fluorescent intensity. If insulin staining was at least 50% more intense than the glucagon intensity, the cell is defined as a β-cell. If glucagon staining was at least 50% more intense than the insulin staining, the cell is defined as an α-cell. If neither signal reaches this 50% greater threshold, the cell is defined as Unclassified. This process was performed in RStudio.

#### Human insulin and C-peptide ELISA and total protein analyses

Human insulin was analyzed with an insulin ELISA kit (Mercodia, 10-1113-10) according to the manufacturer’s instructions. Secretion samples were diluted 1:12.5 in SAB, and insulin content samples were diluted 1:1250 in SAB. C-peptide measurements were done using an ELISA kit (Mercodia, 10-1136-01) according to the manufacturer’s instructions with a sample dilution of 1:5 in SAB. Total protein was quantified in undiluted sample lysates with a BCA assay kit (Pierce, 23225) according to the provided instructions by the manufacturer.

#### Human IGFBP7 ELISA

Human IGFBP7 was analyzed in cell culture medium from EndoC-βH1 cells and islet lysates using an IGFBP7 ELISA kit (BoosterBio, EK0991) according to the provided protocol. Both medium samples and lysates were run undiluted.

#### Viability assessment with MTS assay

Cell viability experiments were performed by seeding EndoC-βH1 cells in 96-well plates and treating them with IGFBP7 at concentrations of 0, 10, 50 and 100 nM for 72 h. Viability assessment was done by adding 20 μL CellTiter MTS assay solution (Promega, G3582) to the cell medium and reading absorbance at 490 nm after a 2-h incubation at 37°C and CO_2_ level 5%.

### Quantification and statistical analysis

#### Statistical analysis

Analysis of RNA-seq data for *IGFBP7* gene expression data presented in Figures 1 and S1 is described in Bacos et al.^19^ HbA1c value was missing in 16 out of 149 available ND donors and these are therefore excluded in Figure 1C. In Figure 1A, these 16 donors are classified as ND. Gene expression and HbA1c spearman correlation analysis and visualization were performed with the R package ggpubr (v0.6.0). Groupwise comparisons of gene expression data and IGFBP7 staining intensity data were done using the Mann-Whitney test. For all functional experiments in human islets and EndoC-βH1 cells, data was plotted in GraphPad prism v. 9 and assessed for normality. For these experiments, statistical tests used were either Student’s T-test or paired T-test for two groups, One- or 2-way RM ANOVA for experiments with multiple groups. Tests used as indicated in figure legends.

## Supplemental information

Document S1. Figures S1, S2, Tables S1, and S2

Table S3. Differential expression analysis of gene changes in human islets after IGFBP7-treatment, related to Figure 2, donors listed in Table S2
