# Supplementary material for: IGFBP7 is upregulated in islets from T2D donors and reduces insulin secretion
Source: iScience. 2024 Aug 20;27(9):110767. doi: 10.1016/j.isci.2024.110767 (PMC11402214; doi:10.1016/j.isci.2024.110767)
Supplement: Document S1. Figures S1, S2, Tables S1, and S2 [file mmc1.pdf]

iScience, Volume *27*

Supplemental information

IGFBP7 is upregulated in islets from T2D donors and reduces insulin secretion

Efraim Westholm, Alexandros Karagiannopoulos, Nicole Kattner, Yara Al-Selwi, George Merces, James A.M. Shaw, Anna Wendt, and Lena Eliasson

Supplementary Figure 1

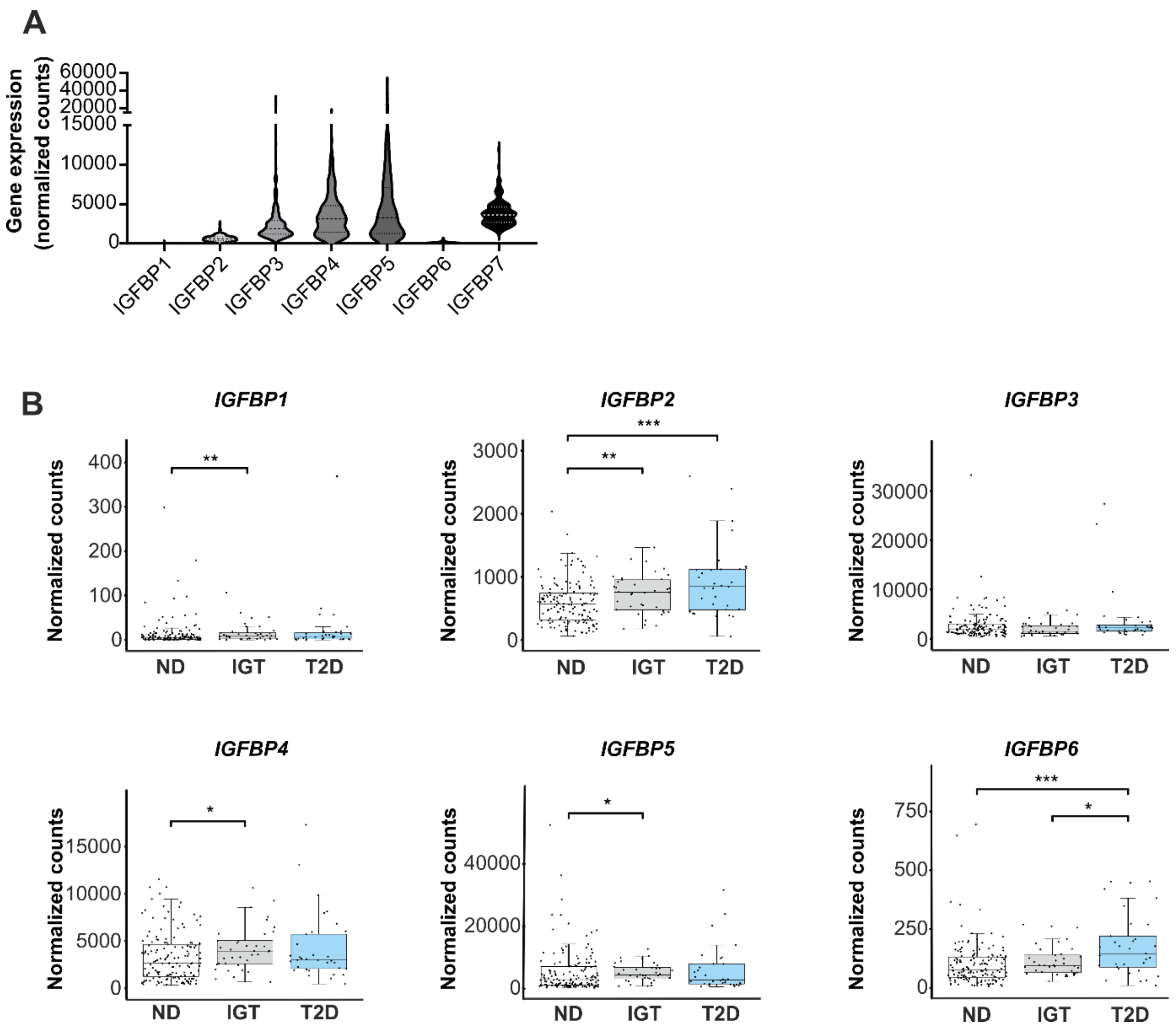

Figure S1: Additional characteristics of IGFBP7 and IGFBP1-6 in human pancreatic islets. Related to Figure 1.

A: Violin graphs for comparison of gene expression of IGFBP1-7 in human pancreatic islets from 219 donors. Dashed lines represent median and 25th and 75th percentiles. **B**: Gene expression of IGFBP1-6 divided by disease state in 219 human donors. Mann-Whitney test in B, the box plots show median and 25th and 75th percentiles. *P<0.05, **P<0.01, ***P<0.001. Data in A and B is from Bacos et al, 20231.

Supplementary Figure 2

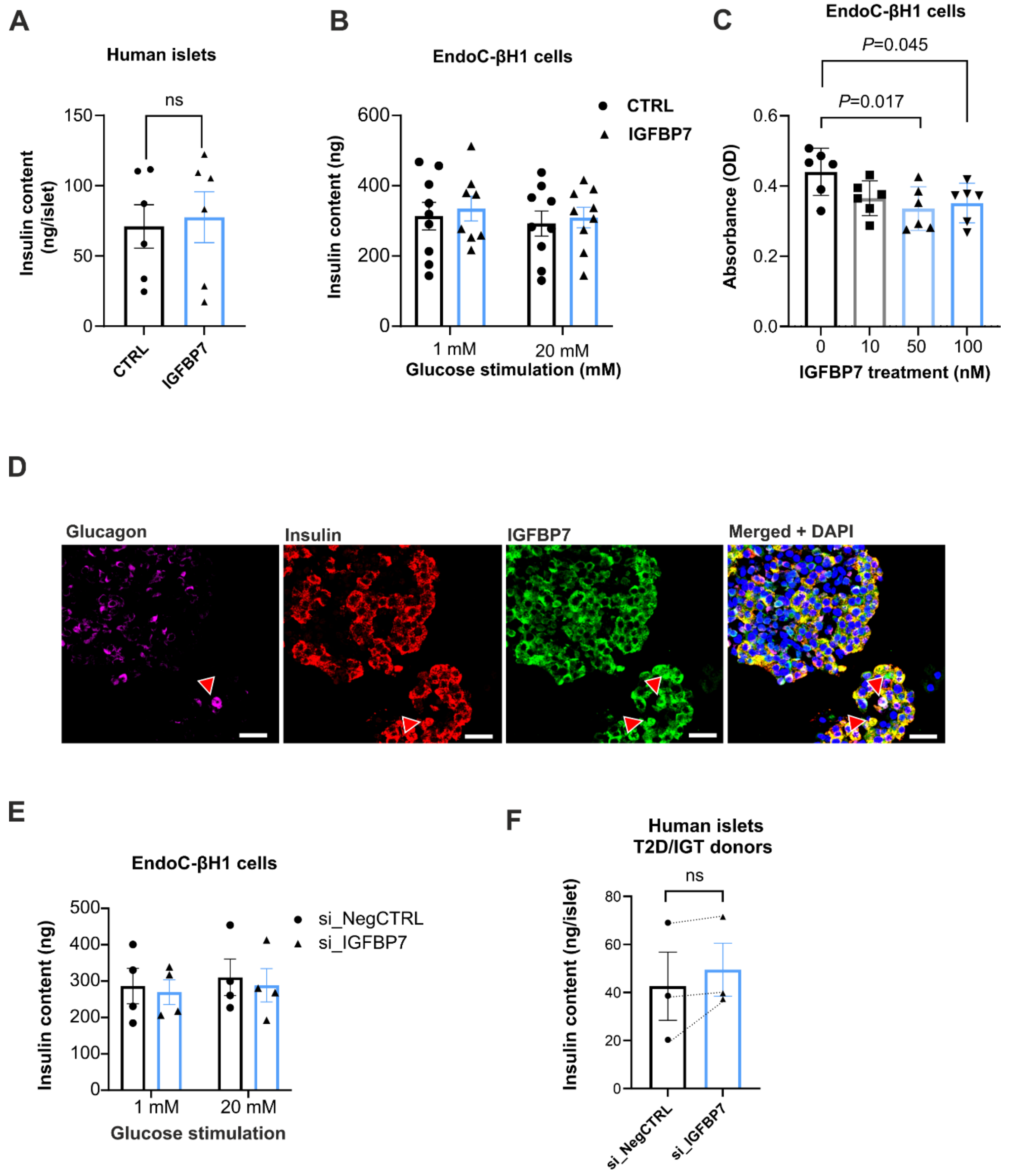

Figure S2: Insulin content is unaffected by IGFBP7 treatment but cell viability is reduced. Panel A related to Figure 2, panel B, C to Figure 3, panel D to Figure 4 and panel E, F to Figure 5.
A: Insulin content in islets from six ND donors after 72-hour IGFBP7 treatment. **B**: Insulin content in nine EndoC-βH1 passages after 72-hour IGFBP7 treatment. **C**: MTS assay in EndoC-βH1 cells after IGFBP7 treatments of 10, 50 and 100 nM. **D**: Immunostaining of glucagon (pink), insulin (red), IGFBP7 (green) and merge with DAPI nuclear stain (blue) in isolated islets from one ND donor. Red arrows show co-localization of IGFBP7 with both glucagon in an α-cell and with insulin in a β-cell. Scale bars: 25 µM. **E**: Total insulin content in EndoC-βH1 cells transfected with si_IBGFP7. **F**: Total insulin content in islets transfected with si_IGFBP7. Paired T-test in A, F, 2-way ANOVA in B and One-way ANOVA in C. Data is presented as mean ±SEM.

Supplementary table 1: Pscan predicted transcription factors for human *IGFBP7*. Related to Figure 1.

Results from Pscan (website URL: http://159.149.160.88/pscan/) for the two mRNA transcript variants of human *IGFBP7* (RefSeq IDs: variant 1 NM_001553, variant 2 NM_001253835). Search constraints: JASPAR 2020_NR database, binding region 450 bp upstream and 50 bp downstream of the gene. Transcription factors with a significant (P<0.05) predicted binding are listed. Search results were obtained on 2024-01-04.

**Rank no.**	**Transcription factor**	***P*-value**
1	RUNX1	0.000766431
2	TBXT	0.00161559
3	ETV2	0.00328117
4	Prdm15	0.00445284
5	Spz1	0.00512686
6	FLI1	0.00521695
7	TBX19	0.00620068
8	POU6F1(var.2)	0.00650447
9	PAX5	0.00657458
10	ERG	0.0111664
11	ETS1	0.0117292
12	Nkx2-5(var.2)	0.0134999
13	ETS2	0.0138053
14	RUNX2	0.0169051
15	ERF	0.0178687
16	POU6F2	0.0185597
17	YY2	0.0225876
18	FEV	0.0294784
19	TFDP1	0.0312221
20	E2F7	0.0314243
21	Zic1::Zic2	0.0327764
22	PLAGL2	0.0332139
23	HES7	0.0366061
24	ELK4	0.0403314
25	HOXB5	0.0424479
26	ETV3	0.0473472

Supplementary table 2: Information on human donors of pancreatic islets and pancreatic tissue slices. Related to Figure 2, Figure 4, Figure 5 and Figure S2. (See table column "Figure" for donor specific information.)

Donor ID	Gender (M/F)	Age	BMI	Disease state (ND/IGT/T2D)	HbA1c (mmol/mol)	HbA1c (%)	Figure	Centre
1	M	62	29,5	ND	40	5,8	2	Lund donors
2	M	55	32,1	ND	38	5,6	2	
3	F	54	18,0	ND	36	5,4	2	
4	F	58	22,5	ND	36	5,4	2	
5	M	46	19,0	ND	34	5,3	2	
6	M	69	27,1	ND	-	-	2	
7	M	48	25,2	ND	40	5,8	4C	
8	M	80	27,7	T2D	45	6,3	5E-G	
9	F	48	28,8	IGT	43	6,1	5E-G	
10	F	50	35,4	IGT	48	6,5	5E-G	
11	F	39	24,3	ND	36	5,4	4B	Newcastle donors
12	F	57	23,2	ND	36	5,4	4B	
13	F	65	23,9	ND	36	5,4	4B	
14	F	70	34,9	T2D	52	6,9	4B	
15	M	49	31,5	ND	40	5,8	4B	
16	F	40	31,6	ND	35	5,4	4B	
17	F	63	32,8	T2D	39	5,7	4B	
18	M	62	28,7	ND	37	5,5	4B	
19	M	40	25,4	T2D	-	-	4B	
20	F	55	23,2	ND	38	5,6	S2D	

References supplemental information

1. Bacos, K., Perfilyev, A., Karagiannopoulos, A., Cowan, E., Ofori, J.K., Bertonnier-Brouty, L., Rönn, T., Lindqvist, A., Luan, C., Ruhrmann, S., et al. (2023). Type 2 diabetes candidate genes, including PAX5, cause impaired insulin secretion in human pancreatic islets. J Clin Invest *133*. 10.1172/jci163612.
